# Astiology of Irregularities of the Jaws and Teeth

**Published:** 1888-05

**Authors:** E. S. Talbot


### THE DENTAL REGISTER.

VOL. XLII.] MAY, 1888. [No. 5.

### COMMUNICATIONS.

### Aetiology of Irregularities of the Jaws and Teeth.

BY E. S. TALBOT, M.D., D.D.S.

By comparing the records of the condition of the teeth of the ancients as determined by the study of existing skulls, together with the teeth of the lower grades of humanity now living, with those of the more civilized races of the present day, it will be observed that irregularities are not only more frequently seen in the latter, but that their frequency increases *pari passu* with advancing civilization. As these deformities are associated with that portion of the human anatomy which we as dental and oral specialists are called upon to treat, they demand our most careful consideration, and an amount of attention which necessitates most thorough observation and research. The maxillary bones develop and grow independently of each other, and therefore one cannot influence the other as regards development or growth. It is quite common to find that the superior maxilla is proportionately much smaller than the inferior, but it is rare that the inferior maxilla is not fully developed. The inferior maxilla, being entirely free from the other bones in the body, develops independently and because of its mobility, constantly receives a large blood supply which amply nourishes it.

The superior maxilla, on the other hand is developed in conjunction with the other bones of the head, consequently becoming one of the fixed bones of the face, and as Mr. Coles suggests, it may be that arrested development of the bone is due to premature ossification of the suture, or according to Cartwright, " to the intermarriage of races and high and select breeding," but personally I entertain the opinion that the arrested development of the superior maxillary bone, is caused by inefficient supply of blood to the part to develop it because of the inaction of the jaw; this is illustrated in other members of the body, exercise or action developing the other part.

Narrow and contracted jaws are more numerous among the present people than was seen in former races. Then they subsisted chiefly upon roots, nuts, grains and coarse foods of all sorts, which required mastication to prepare them for digestion; the jaws were thoroughly exercised and developed accordingly. Now the food is about ready for digestion when taken into the mouth, and mastication is becoming one of the lost arts; the jaws being but little exercised, which frequently result in narrow and contracted arches. Anatomists in describing the maxillary bones speak of them as the superior and inferior maxillæ. But we as orthodontologists should divide each bone into two parts. The parts below the mental foramen on the lower jaw, and above the palate on the upper jaw are hard and dense and are designed for the attachment of muscles. The alveolar processes situated above the mental foramen on the lower jaw, and below the palate on the upper, are expressly for the formation of the teeth while in their crypts, and for their retention after they have erupted. When the teeth are removed the processes are absorbed away, and nothing remains but the dense bone. In intra-uterine life while the teeth are forming, the alveolar processes cover and protect the crypts in which the teeth are located, and as they grow and force their way through the process, absorption takes place and much of the bone vanishes. After the teeth have erupted, deposition of bone again takes place, which holds the teeth firmly in place. Again, these teeth are shed and bone is absorbed to admit the second set, after which new material is deposited for their retention.

It will be observed that from the time the first teeth make their appearance until the second set are firmly fixed in position, the alveolar process has changed twice. These changes, however, do not effect the shape of the maxillary bones proper; the teeth grow and develop independent of the alveolar process, but the processes are dependent upon the teeth for their development, position and shape. If the crypts containing the crowns of the teeth be situated on the inner border of the alveolar process, or if the teeth should erupt through the inner border of the alveolar process, when dentition is completed the diameter of the circle would be much smaller than that of the jaw bone. If, on the contrary, the teeth are situated upon the outer border of the alveolar process and the teeth in their transit are directed outward, the diameter of the circle will be as great or greater than the jaw bone. It may be observed that when several teeth are missing upon one jaw, the teeth of the opposite jaw having no resistance, the alveolar process elongates showing that the physiological process of growth furnishing support to the teeth, is properly performed, though many times under difficulties.

The teeth of the present generation decay more rapidly than those of former times, and are extracted earlier in life. Thus as generations pass, the teeth gradually decrease in the time they remain in the jaw, and the diameter of the alveolar process is reduced, like other parts of the body when deprived of support. The eruptive fevers common among children, or constitutional disturbances of any nature may cause arrested development of the maxillary bones.

Arrested development of the maxillary bones is characteristic of idiotic and feeble-minded children. This fact was emphasized by Dr. Langdon Down in a paper read before the Odontological Society of London in 1871. He said that in examination of a great many congenital idiots he found that with very few exceptions, the arches were contracted in width between the bicuspids, and that irregularity in arrangement of the teeth was the rule rather than the exception. Dr. W. W. Ireland found that out of 81 idiots examined 37 had either vaulted or V-shaped arches.

These opinions awakened the interest of many scientific men of our country; among them were Dr. Kingsley, of New York, and Drs. J. W. White and Stellwagen, of Philadelphia. Dr. Kingsley observed that in the 200 idiots of different nationalities at the Asylum on Randall's Island, "he did not find a single pronounced case of V-shaped dental arch, but very few cases of narrow palatine arch, and but three or four saddle-shaped palates. Drs. J. W. White and Stellwagen examined the inmates of a large school for feeble-minded children in Pennsylvania and found large well shaped jaws the rule. In these reports no definite statistics of the proportion of regular and irregular jaws and teeth are given. It is rather curious that these gentlemen equally eminent on their respective sides of the ocean should differ so widely on this question. In the different institutions for feeble minded, the inmates of which I have examined and from blank reports filled out by resident dentists, of institutions which I could not visit, I deduce the following statistics:

### Tables of Classification of Irregularities of the Jaws and Teeth of Feeble Minded Imbeciles and Idiots in the various Institutions in the United States.

Table 1. Asylum for Idiots of the State of New York, Syracuse, Dr. Casson, Superintendent. Examination made by Drs. S. B. Palmer and J. C. House.*

### DEFORMITIES IN THE JAWS.

### LOW GRADE.

| No. | Sex. | Normal. | Large Jaw. | Protrusion Lower Jaw. | Protrusion Upper Jaw. | High Arch. | V Shaped Arch. | Partial V Shaped Arch. | Thumb Sucking. | Saddle Shaped Arch. | Small Teeth. |
|---|---|---|---|---|---|---|---|---|---|---|---|
| | Male. | | | | | | | | | | |
| | Female. | | | | | | | | | | |
| 160 | | 142 | | | | | 4 | 14 | | | |

* This report is not complete. The examination will be made again.

Table 2. Massachusetts School for Feeble Minded, South Boston, Asbury S. Smith, Superintendent. Examination made by Dr. C. E. Estabrook.

### DEFORMITIES IN THE JAWS.

HIGH GRADE.

| No. | Sex. | Normal. | Large Jaw. | Protrusion Lower Jaw. | Protrusion Upper Jaw. | High Arch. | V Shaped Arch | Partial V Shaped Arch. | Thumb Sucking. | Saddle Shaped Arch. | Small Teeth. |
|---|---|---|---|---|---|---|---|---|---|---|---|
| 23 | Male. | 6 | 5 | 1 | 2 | 15 | 3 | 14 | | 5 | 4 |
| 26 | Female. | 9 | 2 | 2 | 1 | 13 | 2 | 4 | | 7 | 8 |
| 49 | | 15 | 7 | 3 | 3 | 28 | 5 | 18 | | 12 | 12 |
| No. | Sex. | | | | MIDDLE GRADE. | | | | | | |
| 15 | Male. | 2 | 4 | | | 10 | 1 | 4 | | 2 | 2 |
| 8 | Female. | | 4 | 1 | 3 | 5 | | 4 | | 2 | 3 |
| 23 | | 2 | 8 | 1 | 3 | 15 | 1 | 8 | | 4 | 5 |
| No. | Sex. | | | | LOW GRADE. | | | | | | |
| 47 | Male. | 3 | 2 | | 3 | 11 | 1 | 14 | | 3 | 1 |
| 48 | Female. | 4 | 6 | 2 | 8 | 11 | | 9 | 1 | 9 | 2 |
| 95 | | 7 | 8 | 2 | 11 | 22 | 1 | 23 | 1 | 12 | 3 |

Table 3. Illinois Asylum for Feeble Minded Children, Lincoln, Dr. Wm. B. Fish, Superintendent. Examination made by Dr. Eugene S. Talbot.

### DEFORMITIES IN THE JAWS.

MIDDLE GRADE.

| No. | Sex. | Normal. | Large Jaw. | Protrusion Lower Jaw. | Protrusion Upper Jaw. | High Arch. | V Shaped Arch. | Partial V Shaped Arch. | Thumb Sucking. | Saddle Shaped Arch. | Small Teeth. |
|---|---|---|---|---|---|---|---|---|---|---|---|
| 198 | Male. | 148 | | 6 | 7 | | 11 | 25 | | 4 | 2 |
| 149 | Female. | 107 | 4 | 1 | 2 | 10 | 6 | 23 | 2 | 3 | 4 |
| 347 | | 255 | 4 | 7 | 9 | 10 | 17 | 48 | 2 | 7 | 6 |

Table 4. Asylum for Idiots, Randalls Island, Dr. Healey, Superintendent. Examination made by Dr. E. J. Ranhofer.

### DEFORMITIES IN THE JAWS.

### HIGH GRADE.

| No. | Sex. | Normal | Large Jaw. | Protrusion Lower Jaw. | Protrusion Upper Jaw. | High Arch. | V Shaped Arch. | Partial V Shaped Arch. | Thumb Sucking. | Saddle Shaped Arch. | Small Teeth. |
|---|---|---|---|---|---|---|---|---|---|---|---|
| 40 | Male. | 11 | 12 | 3 | 15 | 24 | 4 | 7 | | 12 | 2 |
| 52 | Female. | 23 | 7 | 5 | 7 | 19 | 5 | | | 6 | |
| 92 | | 34 | 19 | 8 | 22 | 43 | 9 | 9 | | 18 | 2 |
| No. | Sex. | | | | MIDDLE GRADE. | | | | | | |
| 9 | Male. | 3 | 1 | 2 | 3 | 5 | | | | 1 | |
| | Female. | | | | | | | | | | |
| 9 | | 3 | 1 | 2 | 3 | 5 | | | | 1 | |
| No. | Sex. | | | | LOW GRADE. | | | | | | |
| 33 | Male. | 7 | 10 | 8 | 18 | 20 | 7 | | | 12 | |
| 23 | Female. | 7 | 3 | 1 | 11 | 17 | 2 | | | 8 | |
| 56 | | 14 | 13 | 9 | 29 | 37 | 9 | | | 20 | |

Table 5. Minnesota Training School for Idiots and Imbeciles, Faribault, Dr. A. C. Rogers, Superintendent. Examination made by Dr. S. T. Clements.

### DEFORMITIES IN THE JAWS.

| No. | Sex. | Normal. | Large Jaw. | Protrusion Lower Jaw. | Protrusion Upper Jaw. | High Arch. | V Shaped Arch. | Partial V Shaped Arch. | Thumb Sucking. | Saddle Shaped Arch. | Small Teeth. |
|---|---|---|---|---|---|---|---|---|---|---|---|
| | | | | | HIGH GRADE. | | | | | | |
| 47 | Male. | 18 | 1 | | 3 | 7 | 1 | 1 | | 18 | |
| 22 | Female. | 12 | 2 | | | | 2 | 1 | 2 | 5 | |
| 69 | | 30 | 3 | | 3 | 7 | 3 | 2 | 2 | 23 | |
| No. | Sex. | | | | MIDDLE GRADE. | | | | | | |
| 20 | Male. | 7 | | 1 | 1 | 2 | | 1 | | 10 | |
| 16 | Female. | 2 | | 1 | 1 | 1 | 1 | 2 | 2 | 11 | |
| 36 | | 9 | | 2 | 2 | 3 | 1 | 3 | 2 | 21 | |
| No. | Sex. | | | | LOW GRADE. | | | | | | |
| 12 | Male. | 3 | 2 | 1 | | | | | 3 | 8 | |
| 11 | Female. | 3 | 1 | | 2 | 1 | | 2 | 4 | 4 | |
| 23 | | 6 | 3 | 1 | 2 | 1 | | 2 | 7 | 12 | |

Table 6. Kansas State Asylum of Idiotic and Imbecile Youths, Winfield, Dr. H. M. Greene, Superintendent. Examination made by the Superintendent.

### DEFORMITIES IN THE JAWS.

HIGH GRADE.

| No. | Sex. | Normal. | Large Jaw. | Protrusion Lower Jaw. | Protrusion Upper Jaw. | High Arch. | V Shaped Arch. | Partial V Shaped Arch. | Thumb Sucking. | Saddle Shaped Arch. | Small Teeth. |
|---|---|---|---|---|---|---|---|---|---|---|---|
| 2 | Male. | | 1 | | | 1 | | | | | |
| 3 | Female. | 2 | | | 1 | 2 | | | | | |
| 5 | | 2 | 1 | | 1 | 3 | | | | | |
| No. | Sex. | | | | MIDDLE GRADE. | | | | | | |
| 11 | Male. | 2 | 1 | 3 | 2 | 3 | 2 | 6 | | | 4 |
| 7 | Female. | 2 | 1 | 2 | | 1 | | 6 | | | 3 |
| 18 | | 4 | 2 | 5 | 2 | 4 | 2 | 12 | | | 7 |
| No. | Sex. | | | | LOW GRADE. | | | | | | |
| 10 | Male. | | 3 | | | 3 | 5 | 9 | | 1 | 4 |
| 12 | Female. | | 1 | 7 | 2 | 1 | 2 | 9 | 1 | 1 | 4 |
| 22 | | | 4 | 7 | 2 | 4 | 7 | 18 | 1 | 2 | 8 |

Table 7. Cook County Insane Asylum, Dunning, Ill., Dr. Spray, Superintendent. Examined by Dr. Eugene S. Talbot assisted by Dr. Bryan and Hunt.

### DEFORMITIES IN THE JAWS.

### LOW GRADE.

| No. | Sex. | Normal. | Large Jaw. | Protrusion Lower Jaw. | Protrusion Upper Jaw. | High Arch. | V Shaped Arch. | Partial V Shaped Arch. | Thumb Sucking. | Saddle Shaped Arch. | Small Teeth. |
|---|---|---|---|---|---|---|---|---|---|---|---|
| 32 | Male. | 10 | 2 | 4 | 1 | 4 | 6 | 8 | 0 | 2 | 2 |
| 17 | Female. | 8 | 3 | 2 | 0 | 3 | 4 | 5 | 0 | 1 | 2 |
| 49 | | 18 | 5 | 6 | 1 | 7 | 10 | 13 | 0 | 3 | 4 |

Table 8. Pennsylvania Institution for Feeble Minded Children, Elwyn, Penn., Dr. J. W. Kerlin, Superintendent. Examination by Dr. Wilmarth.

### DEFORMITIES IN THE JAWS.

HIGH GRADE.

| No. | Sex. | Normal. | Large Jaw. | Protrusion Lower Jaw. | Protrusion Upper Jaw. | High Arch. | V Shaped Arch. | Partial V Shaped Arch. | Thumb Sucking. | Saddle Shaped Arch. | Small Teeth. |
|---|---|---|---|---|---|---|---|---|---|---|---|
| 113 | Male. | 80 | 2 | 1 | 1 | 14 | 3 | 8 | 2 | 3 | 1 |
| 72 | Female. | 55 | 0 | 1 | 0 | 6 | 2 | 4 | 3 | 2 | 0 |
| 185 | | 135 | 2 | 2 | 1 | 20 | 5 | 12 | 5 | 5 | 1 |
| No. | Sex. | | | | MIDDLE GRADE. | | | | | | |
| 121 | Male. | 84 | 0 | 3 | 0 | 12 | 7 | 3 | 1 | 9 | 3 |
| 94 | Female | 72 | 0 | 1 | 0 | 2 | 5 | 8 | 1 | 3 | 2 |
| 215 | | 156 | 0 | 4 | 0 | 14 | 12 | 11 | 2 | 12 | 5 |
| No. | Sex. | | | | LOW GRADE. | | | | | | |
| 80 | Male. | 49 | 1 | 2 | 2 | 8 | 8 | 3 | 4 | 3 | 2 |
| 72 | Female. | 43 | 1 | 2 | 1 | 7 | 5 | 6 | 5 | 4 | 1 |
| 152 | | 92 | 2 | 4 | 3 | 15 | 13 | 9 | 9 | 7 | 3 |

Table No. 9. Total number of Deformities of the Jaws of both sexes in each Grade.

### DEFORMITIES IN THE JAW.

HIGH GRADE.

| No. | Sex. | Normal. | Large Jaw. | Protrusion Lower Jaw. | Protrusion Upper Jaw. | High Arch. | V Shaped Arch. | Partial V Shaped Arch. | Thumb Sucking. | Saddle Shaped Arch. | Small Teeth. |
|---|---|---|---|---|---|---|---|---|---|---|---|
| 225 | Male. | 115 | 21 | 5 | 21 | 61 | 11 | 30 | 2 | 38 | 7 |
| 175 | Female. | 101 | 11 | 8 | 9 | 40 | 13 | 9 | 5 | 20 | 8 |
| 400 | | 216 | 32 | 13 | 30 | 101 | 24 | 39 | 7 | 58 | 15 |
| No. | Sex. | | | | MIDDLE GRADE. | | | | | | |
| 374 | Male. | 246 | 10 | 15 | 13 | 32 | 21 | 39 | 1 | 26 | 11 |
| 274 | Female. | 183 | 10 | 6 | 6 | 19 | 12 | 43 | 5 | 19 | 12 |
| 648 | | 429 | 20 | 21 | 19 | 51 | 33 | 82 | 6 | 45 | 23 |
| No. | Sex. | | | | LOW GRADE. | | | | | | |
| 214 | Male. | 72 | 20 | 15 | 24 | 46 | 27 | 34 | 7 | 29 | 9 |
| 343 | Female. | 207 | 15 | 14 | 24 | 40 | 13 | 45 | 11 | 27 | 9 |
| 557 | | 279 | 35 | 29 | 48 | 86 | 40 | 79 | 18 | 56 | 18 |

Table No. 10. Total number of deformities of the jaw of both sexes in all grades.

### DEFORMITIES IN THE JAWS.

| No. Total. | Sex. | Normal. | Large Jaw. | Protrusion Lower Jaw. | Protrusion Upper Jaw. | High Arch. | V Shaped Arch. | Partial V Shaped Arch. | Thumb Sucking. | Saddle Shaped Arch. | Small Teeth. |
|---|---|---|---|---|---|---|---|---|---|---|---|
| 1605 | Male. | 924 | 87 | 63 | 97 | 238 | 101 | 200 | 31 | 159 | 56 |
| Per cent. | | 57.7 | 5.4 | 4.9 | 6.0 | 13.5 | 6.2 | 11.4 | 1.9 | 9.9 | 4.4 |

It must be conceded that in an equal number of strong and feeble-minded persons the larger percentage of irregularities is found in the latter class. These irregularities do not confine themselves to the V and saddle-shaped arches, but statistics show a large percentage of partial V-shaped arches and arrested development of the maxillary bone. Excessive growth of the superior maxilla and protruding superior and inferior jaws also share in the generally irregular character of the jaws and teeth of the feeble-minded.

In the examination of the mouths of idiots the class distinction shows itself very conspicuously. The three grades, high, medium and low bred idiots, carry the stamps of their respective social positions in the shape of the jaws quite as prominently as other denominating characteristics. Institutions containing either class exclusively, will record the characteristic jaws of that special grade; consequently examinations by different men in different institutions may not show the same proportion of cases of a given deformity. Irregularities of individual teeth and those caused by extraction of, or want of attention to, the deciduous teeth were not recorded, although they were numerous.

The high, the V, and the saddle-shaped arches mentioned so frequently by Drs. Down, Ireland and Ballard as being common among foreign idiots, are seen as often among the idiots of our own country. But the percentage of thumb-sucking cases is so small, that it does much to disprove the *reductio ad absurdum* that thumb-sucking precedes, and causes idiotcy—if indeed any disproval be necessary.

HEREDITY IN ITS RELATIONS TO IRREGULARITIES.

It is a fact universally recognized that various morbid conditions and peculiarities of structure are often transmitted from parent to child, through many generations. This law of heredity is almost universal in its application, and its influence may be either enhanced or depreciated through successive or alternate generations, until we have upon the one hand a total disappearance of the heredity impression, or upon the other, an increase so great that the condition becomes incompatible with the life of the individual. This variation is a fortunate circumstance, as by it the human race is protected from certain destruction.

This plan of variation is powerful for good or evil, according to the environment of the individual, or of the family to which he belongs. This fundamental evolutionary law of heredity is nowhere more manifest than in the case of perversion of development of both internal and external organs, either embryonal or post natal, and it is a most powerful factor in the production of deformities of the jaw and irregularities of the teeth. Not only does this hold true in the case of general irregularities due to maxillary deformities, but it also applies to malformations of individual teeth. Thus I have observed in a family consisting of mother, daughter and grand daughter, a peculiar fissured condition of the enamel upon the labial surface of a left superior lateral incisor.

It is not uncommon for a child to possess peculiarities of the teeth of one jaw, resembling those present in the father, while the other presents irregularities of development precisely identical with those present in the mother; again, one parent may transmit peculiarities of maxillary development, while the other transmits certain characteristic appearances of the teeth. Much has been said of late regarding the influence of ante-natal impressions, upon the development of deformities, and if the claims advanced be but half true, it is probable that the teeth and jaws may occasionally suffer their share of the resulting detriment. Evidence of dental deformities from this cause is of necessity difficult to obtain. A case is recalled, however, in which a peculiar condition of irregularity of teeth was attributed by the mother to her constant worry during gestation lest the coming child should have teeth as irregular as her own. When dentition was finally completed in the child, the arrangement of the teeth was identical with those of the mother. This case is not advanced as an evidence of ante-natal impressions, but because of its suggestiveness.

Very often we are absolutely unable to determine the precise degree of influence exerted by heredity, even when we are convinced that it is a powerful factor. It is evident to any one upon reflection, that the same causes which will produce deformities independent of hereditary influences will also prevent the latter from acting as they otherwise would.

The teeth are creatures of circumstances, *i. e.* they develop independently of the alveolar process, hence their order of development and the resistance imparted by other teeth and roots all combine to produce irregularities, in short local causes produce a majority of irregularities and modify formations which might be otherwise the exact counterpart of those presented by the teeth of the parent.

The following cases in practice illustrate this theory. In one family under my observation, the father's jaws are well developed, and contain large strong teeth. The mother's jaws are small, the teeth being regular in the lower maxilla. In the upper maxilla the central incisors are regular and in normal position, but the canines, bicuspids, and molars have come forward and filled the spaces occupied by the laterals, which were extracted at the age of thirteen. Two sons (their only children,) have lower jaws and teeth closely resembling the mother's. The upper jaws and teeth of both resemble the father's in size and strength, but unlike the father's they are very irregular in position. These irregularities are not due to limited space in the jaws, which are sufficiently large to admit the teeth with regularity. This tendency to irregularity of position is a marked inheritance from the mother. In the case of the eldest son, who is fourteen years of age the central incisors of the upper jaw are regular, the laterals are forced by the cuspids some distance inside the natural line, the cuspids, bicuspids and molars are anterior to their normal position. The second case illustrates the jaws of the younger son, aged eleven. The centrals and laterals erupted at the proper time. The cuspids are encroaching upon them to such an extent as will eventually form a V-shaped arch. Both boys have been under my care from the beginning, the temporary teeth being removed at the proper time.

It will be observed that the tendency toward irregularity in arrangement is decidedly inherited from the mother. The conditions are so modified by local influences that although the hereditarily irregular arrangement comes from the mother, they are not exact counterparts of the mother's irregularity, nor are they alike. It is questionable whether exact counterparts of irregularities are ever inherited from parents. Various local interferences and conditions will, as we have seen, influence this one way or the other. Transmissions of small jaws and of pecuiarities of individual teeth are, however, quite common.

In 1864 Messrs. Cartright & Coleman, of London, examined some 200 skulls in the crypt of Kythe church, Kent, which had been deposited there for centuries. They found the alveolar processes and teeth perfectly developed and formed.

In 1869 Mr. John R. Mummery, London, read a paper before the Odontological Society of Great Britain in which he gave a report of his extended researches including over 3,000 skulls of ancient and modern uncivilized races, and concluded that the early and half savage people were freer from dental irregularities than moderns. Dr. Nichols, of New York, has examined the mouths of thousands of Indians and Chinese, and says with but one exception he never found an instance of irregularities in either of these races.*

I can confirm the statement of Dr. Nichols as regards the Chinese, having examined the teeth of many of them on the Pacific Coast. The above reports, together with the testimony of other investigators, show that ancient, uncivilized and nomadic barbarians have perfectly shaped dental arches.

The interesting circumstance that irregularities occur more frequently now than formerly, and among people living in new countries would suggest the idea that irregularities caused by heredity may result from the intermarriage of different nationalities; the offspring of such unions partaking irregularly and in different degrees, of the racial peculiarities of maxillary development of either or both parents. It is probable that the varying character of food, and the abuse of the teeth incident to the depraved hygiene of modern civilization have much to do with dental malformations. Again, the higher the evolutionary type of individuals, the more imperfect the teeth and jaws become. The nearer the monkey, and the farther removed from refined and civilized man, the better the teeth. As the animal becomes less and less dependent upon his jaws and teeth for a livlihood, the

* Kinsley's Oral Deformities.

less perfect these structures become, and after the lapse of many generations, marked variations and imperfections of development are logically to be expected.

IRREGULARITIES THE RESULT OF ARRESTED DEVELOPMENT.

As a result of arrested development two forms of irregularities are observed ; the V-shaped and the saddle-shaped arches. A similar result follows the too early extraction of the temporary molars, the modus operandi of which will be given further on. The normal size of the teeth vary but slightly from those of former ages, when deformities of the jaws, or irregularities of the teeth did not exist. We must therefore look for the cause of these deformities in the jaw and its relation to the development of the teeth.

The V shaped arch is always associated with the superior maxilla, never with the inferior. It is not associated with the first set of teeth, as the jaws always change after the eruption of the first permanent molars and incisors. The central incisors are turned in their sockets so that their mesial surfaces are directed towards the lip, the crowns inclining forward and carrying the alveolar process with them. The lateral incisors have changed their positions posteriorly, towards the median line, not to the extent, however, of the centrals. The cuspids may be situated on a line with the laterals and bicuspids, but are frequently partially or wholly outside. The bicuspids and first molars have usually dropped forward and become directed inward, until all, or nearly all of the space usually occupied by the cuspid is filled. The roof of the mouth may or may not be vaulted, but the teeth are always crowded, in this deformity. According to Dr. Tomes this malformation is associated with greatly enlarged tonsils, which nessitates breathing through the open mouth.

Dr. Kingsley thinks that it is nearly always of congenital origin, that it is an inherited tendency, favored in all probability by like circumstances with those which initiated it, in the ancestry. Mr. Oakley Coles is of the opinion that premature ossification of the sutures produces this deformity.

Mr. Cartwright attributes the deformity to high breeding and luxurious habits of life in the ancestry of the subject.

Dentists commonly class it as one of the results of thumb-sucking, one author speaking of it as a thumb-sucking variety of deformity. Dr. Ballard, of England, after his investigations of the subject, discovered the V-shaped arch to prevail with idiots and held the theory that thumb-sucking was a forerunner of idiocy, thus applying the argument of *post hoc ergo propter hoc* with a vengeance.

Peculiarities in the size and shape of the jaw bone may be inherited, but the manner of the eruption of the teeth can not be transmitted. The jaw is always small in the V shaped arch, and the first permanent molars appear and have large long roots which establish them as fixed points of resistance. At the anterior part of the mouth, the next teeth to erupt are the central incisors, which, with their round conical roots and broad crowns, make a perfect wedge. The laterals are next to follow, and are smaller than the centrals, but with much the same appearance. These teeth are situated in a long thin alveolar process, which with the broad cutting edges and long conical roots of the central incisors, offers but slight resistance to the pressure between the laterals and the bicuspids and first permanent molars. Next come the first and second bicuspids into place. The last to erupt are the cuspids, which with their long roots and their situation outside the arch possess great leverage. They are propelled downward by the pressure of the lips, and guided by the roots of the lateral and first bicuspid teeth. If the space is small, they will encroach upon the lateral incisors in as much as they are smaller than the first bicuspids, and will force them (the incisors) forward and inward.

The point of contact would naturally be upon the crowns, and as a result the cutting edges of the incisors are carried forward, and being conical, are rotated in their sockets. While this is going on externally, the tongue, owing to the narrow space between the first molars, is contracted laterally and necessarily elongated; the tongue being largely instrumental in shaping the contour of the alveolar process, and in fixing the position of the teeth. When the temporary cuspids and molars are prematurely removed from well developed jaws, the bicuspids and permanent molars work forward and fill the space, and the V-shaped arch will be formed in the manner just described. In this deformity the hard palate may be either low or vaulted.

The saddle-shaped jaw is usually found in connection with the superior maxilla, although it is occasionally found in the inferior maxilla. It will be observed that in this deformity the central incisors, unlike those in the V-shaped deformity, never protrude, but always stand vertical with the alveolar process. The teeth are always close together, the alveolar process is much smaller in diameter than the body of the jaw, and the alveolar process is situated on the inner border of the bone, being directed inward at the bicuspid and first molar region, and outward at the second and third molars. The cuspids may either be in their normal positions, or pushed forward and outward, and in common with the incisors and alveolar process on the lower jaw they present a straight line.

This deformity has its primary cause in the location of the crowns of the permanent teeth in a dwarfed alveolar process. The bicuspids and molars instead of being in the jaws in their normal position stand with their crowns inclined to the roof of the mouth. This abnormal position in the jaws may represent a natural position of the follicles, or the bicuspids may be deflected by roots of the temporary teeth, and the crowns directed towards the roof of the mouth. When the permanent teeth erupt, the order is changed, the centrals and laterals take the natural position, but the cuspids follow instead of the bicuspids. These teeth make a fixed point of resistance in the anterior part of the mouth, thus protecting the incisors and preventing their protrusion. The first permanent molars work forward and become a fixed point in the posterior part of the mouth. The space between the first molar and cuspid is smaller than the long diameter of the crowns of the bicuspids and both are crowded in towards the roof of the mouth. The lateral and forward pressure is so great that with the aid of the tongue the cuspids are carried forward and outward until they and the alveolar process appear flattened. It sometimes happens that the first bicuspid erupts and secures its position before the second bicuspid appears. In this case, the crown of the first permanent molar will come in contact with the crown of the second bicuspid and will form an inclined plane, thus carrying the second bicuspid inside the arch, which will frequently be found turned in its socket, the cusps facing the anterior and posterior part of the mouth. In all cases the alveolar process is built about the teeth, giving them strength and firmness in whatever position they assume.

This deformity is always associated with a high vaulted arch. It is less frequently seen than the V-shaped arch because the natural order of eruption is interfered with in the saddle shaped deformity; the cuspids appearing before the bicuspids. We find in the saddle shaped arch that the second and third molars are forced laterally. The alveolar process is carried with it and rounded out. The same condition is also noticed in jaws having very flat arches. These abnormal conditions are caused by the pressure of the tongue against the posterior part of the hard palate; in the process of swallowing, the constricted arch preventing the normal position of the tongue.

### THUMB-SUCKING.

As compared with other causes, thumb-sucking but rarely produces irregularities of the teeth and jaws. Those irregularities which are produced by this vicious habit are not uniform or extensive. They may occur in the center of the jaw or upon either side, this depending upon the position of the thumb or finger. The teeth of either jaw may be prevented from erupting or the alveolar process may not develop on account of the pressure. The superior teeth and jaw may be brought forward by absorption and deposition of bone, and the lower teeth and alveolar process carried backward in the same manner, by the pressure of the thumb. The inferior maxilla may be carried backward, in which event the angle will be a right angle instead of an obtuse one. In thumb-sucking the arch changes to an oval shape rather than to a sharp V shaped angle. The habit of thumb sucking is acquired in infancy and continued while the first teeth are in the jaw, when the roots are small and very impressible. It will be found that this form of irregularity does not occur in the second set of teeth unless the habit of thumb-sucking was acquired and made manifest in the first set of teeth, while the alveolar process was in a constructive and formative stage and easily influenced by pressure in any direction. During this time the teeth also are readily moved by constant pressure of the thumb, finger, or lip. Such irregularities of the first set of teeth are easily diagnosed. The rapid growth of the jaws and the comparatively small teeth at this period would admit of no other general deformity. The habit is usually outgrown or corrected by the time the permanent teeth appear. If the habit is continued after the second teeth erupt, they will assume the shape and position of their predecessors. The teeth protrude in a fan shape which is quite marked, on account of the anterior teeth being large and long; the alveolar process meantime will take the shape of the object sucked, making it round instead of angular. There are always spaces between the teeth of the superior maxilla, a characteristic not seen in other irregularities. The teeth of the inferior maxilla are always crowded inward. Occasionally the finger or substance sucked is carried far enough into the mouth to change the shape of the alveolar process, or the palatine surfaces of the incisors, but the hard palate is seldom changed by such pressure. The arch or hard palate in such cases, is as likely to be perfectly flat as it is to be vaulted. In the following case taken from a model in the collection of Dr. E. D. Swain, of Chicago, the deformity is exactly in the center of the superior maxilla, involving the incisors and canines. The hard palate is flat in the extreme, consequently the thumb might have produced absorption of the bones, or a change in the shape of the arch, were the prevailing theory correct, but in this case no change took place.

